# Moving towards a taxonomy of cognitive impairments in epilepsy: application of latent profile analysis to 1178 patients with temporal lobe epilepsy

**DOI:** 10.1093/braincomms/fcac289

**Published:** 2022-11-04

**Authors:** Anny Reyes, Bruce P Hermann, Robyn M Busch, Daniel L Drane, William B Barr, Marla J Hamberger, Scott C Roesch, Carrie R McDonald

**Affiliations:** Center for Multimodal Imaging and Genetics, University of California San Diego, La Jolla, CA 92093, USA; San Diego State University/University of California San Diego Joint Doctoral Program in Clinical Psychology, San Diego, CA 92120, USA; Department of Neurology, University of Wisconsin School of Medicine and Public Health, Madison, WI 53726, USA; Epilepsy Center, Neurological Institute, Cleveland Clinic, Cleveland, OH 44106, USA; Department of Neurology, Cleveland Clinic, Cleveland, OH 44195, USA; Department of Neurology, Emory University School of Medicine, Atlanta, GA 30322, USA; Department of Pediatrics, Emory University School of Medicine, Atlanta, GA 30322, USA; Department of Neurology, University of Washington, Seattle, WA 98195, USA; Department of Neurology, NYU-Langone Medical Center and NYU School of Medicine, New York, NY 10016, USA; Department of Psychiatry, NYU-Langone Medical Center and NYU School of Medicine, New York, NY 10016, USA; Department of Neurology, Columbia University, New York, NY 10027, USA; Department of Psychology, San Diego State University, San Diego, CA 92182, USA; Department of Psychiatry, University of California San Diego, La Jolla, CA 92093, USA; Department of Radiation Medicine and Applied Sciences, University of California San Diego, La Jolla, CA 92093, USA

**Keywords:** temporal lobe epilepsy, cognitive phenotype, taxonomy, latent profile analyses, data-driven approach

## Abstract

In efforts to understand the cognitive heterogeneity within and across epilepsy syndromes, cognitive phenotyping has been proposed as a new taxonomy aimed at developing a harmonized approach to cognitive classification in epilepsy. Data- and clinically driven approaches have been previously used with variability in the phenotypes derived across studies. In our study, we utilize latent profile analysis to test several models of phenotypes in a large multicentre sample of patients with temporal lobe epilepsy and evaluate their demographic and clinical profiles. For the first time, we examine the added value of replacing missing data and examine factors that may be contributing to missingness. A sample of 1178 participants met the inclusion criteria for the study, which included a diagnosis of temporal lobe epilepsy and the availability of comprehensive neuropsychological data. Models with two to five classes were examined using latent profile analysis and the optimal model was selected based on fit indices, posterior probabilities and proportion of sample sizes. The models were also examined with imputed data to investigate the impact of missing data on model selection. Based on the fit indices, posterior probability and distinctiveness of the latent classes, a three-class solution was the optimal solution. This three-class solution comprised a group of patients with multidomain impairments, a group with impairments predominantly in language and a group with no impairments. Overall, the *multidomain* group demonstrated a worse clinical profile and comprised a greater proportion of patients with mesial temporal sclerosis, a longer disease duration and a higher number of anti-seizure medications. The four-class and five-class solutions demonstrated the lowest probabilities of a group membership. Analyses with imputed data demonstrated that the four-class solution was the optimal solution; however, there was a weak agreement between the missing and imputed data sets for the four-Class solutions (κ = 0.288, *P* < 0.001). This study represents the first to use latent profile analysis to test and compare multiple models of cognitive phenotypes in temporal lobe epilepsy and to determine the impact of missing data on model fit. We found that the three-phenotype model was the most meaningful based on several fit indices and produced phenotypes with unique demographic and clinical profiles. Our findings demonstrate that latent profile analysis is a rigorous method to identify phenotypes in large, heterogeneous epilepsy samples. Furthermore, this study highlights the importance of examining the impact of missing data in phenotyping methods. Our latent profile analysis-derived phenotypes can inform future studies aimed at identifying cognitive phenotypes in other neurological disorders.

## Teaser

Reyes *et al*. utilized latent profile analyses to derive cognitive phenotypes in 1178 patients with temporal lobe epilepsy. The authors found that the three-phenotype model was the most meaningful based on several fit indices, was the most robust to missing data and produced phenotypes with unique demographic and clinical profiles.

## Graphical Abstract

**Graphical abstract:**
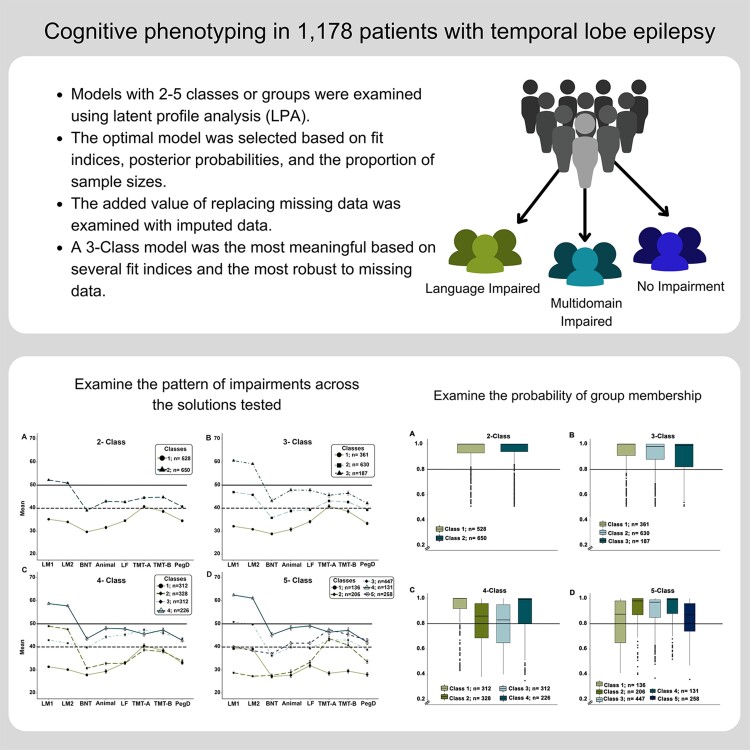

## Introduction

The cognitive comorbidities of epilepsy have been an area of research inquiry for over a century^1^ and are now part of the formal definition of epilepsy.^2^ Historically, the lesion model has been used to examine the relationship between epilepsy pathology and cognition, yielding syndrome-specific cognitive profiles.^3^ However, a myriad of studies have demonstrated that cognitive impairments in epilepsy are more widespread and generalized than hypothesized by the lesion model.^4,5^ For example, patients with temporal lobe epilepsy (TLE) demonstrate impairments in domains that are not typically associated with temporal lobe damage (i.e. executive function) and those with frontal lobe epilepsy demonstrate impairments in ‘non-frontal lobe’ functions (i.e. memory).^6–9^ Further, there is significant variability within epilepsy syndromes with some patients demonstrating generalized impairment while others have minimally impaired profiles despite having similar clinical features.^3,8,10–12^

In efforts to better understand the cognitive heterogeneity within and across epilepsy syndromes, an emerging taxonomy has been proposed and validated in several independent samples. The phenotyping approach identifies *latent groups* or *phenotypes* that share similar patterns of performance across a series of neuropsychological tests. To date, 18 studies have identified phenotypes based on objective or subjective cognitive impairments across a range of epilepsy disorders (for a review, see Hermann *et al*.).^3^ Several of these studies have also found neuroimaging correlates that are unique to each phenotype and more directly map onto the pattern of cognitive impairment that is otherwise obscured by the lesion-based model.^11,13–16^ Furthermore, this approach has been shown to be useful in examining cognitive progression^10^ and post-operative cognitive decline.^17^ Importantly, this new taxonomy allows for the integration of non-epilepsy factors that are known to impact cognition and exacerbate existing neurological disorders and may further explain the heterogeneity in cognitive impairment observed within epilepsy syndromes.^3^ Although cognitive phenotypes are research-based at present and the purpose is not to promote clinical use, phenotyping offers researchers a new approach to examine the underlying neuroanatomical correlates associated with common patterns of cognitive impairments, explore how these phenotypes are associated with progression and post-operative cognitive outcomes and develop a harmonized approach to cognitive classification in epilepsy research.

In TLE specifically, three to five phenotypes have been identified, with three consistent groups across studies: a group of patients with generalized impairment, a group with a more domain-specific profile (e.g. memory and language) and a subgroup with minimally impaired cognitive profiles.^10–13,15,18,19^ The generalized and intact phenotypes have been uniformly described across studies; however, there has been substantial variability in the number and nature of the ‘focal’ or domain-specific phenotypes across investigations. Thus, a final taxonomy remains to be determined. The variability in the domain-specific group may in part be due to differences in the methodology used across investigations, the neuropsychological battery used and the characteristics of the sample. Methods for cognitive phenotyping have included data-driven approaches such as cluster analysis as well as actuarial approaches, which consist of establishing and applying *a priori* criteria for impairment. Our group has demonstrated that there is high concordance between phenotypes derived from cluster analysis and actuarial neuropsychology criteria; however, cluster analysis tends to misclassify patients with clinically defined cognitive impairments as having intact cognition.^12^ Furthermore, many of the phenotype studies have been conducted in single epilepsy centres with modest sample sizes, which could have impacted the number and nature of the derived phenotypes. We argue that studies with large samples and rigorous methodology are needed in order to derive a definitive taxonomy, particularly as we aim to translate these research-based phenotypes into clinical practice or deploy our model for international use.

The utility of the cognitive phenotyping approach has been evaluated in other neurological, developmental and psychiatric disorders, including multiple sclerosis,^20,21^ Parkinson’s disease,^22,23^ autism spectrum disorder^24^ and childhood psychiatric disorders.^25^ These studies have demonstrated that deriving more clinically meaningful cognitive phenotypes rather than aggregating patients by their condition leads to a better understanding of the pathophysiological mechanisms underlying these conditions. For example, De Meo *et al*.^21^ identified five phenotypes in a large sample of patients with multiple sclerosis and examined the clinical and neuroimaging features of each cognitive phenotype. The authors found that each phenotype was characterized by unique clinical and neuroimaging features. Patients with preserved cognition had less severe disability and circumscribed neuroimaging findings, while those with ‘severe-multidomain’ impairment had widespread brain abnormalities. The authors emphasized that identifying cognitive phenotypes in multiple sclerosis will allow a better selection of candidates for cognitive rehabilitation trials. Given that cognitive phenotyping is a patient-centred approach (i.e. cognitive classification is based on the patient’s cognitive profile), it could eventually inform personalized treatments for a variety of neurological, psychiatric and developmental disorders, such as the development of clinical trials aimed at reducing the impact of these conditions on cognitive outcomes.

Although several phenotype models have been reported in the epilepsy literature, this represents the first study to use latent profile analyses (LPA) to consider and compare multiple models. LPA is a person-centered statistical technique that classifies individuals into groups based on their patterns of responses to a set of observed variables.^26,27^ The primary goal of LPA is to maximize both the homogeneity within groups and the heterogeneity between groups. The selection of the optimal number of groups or *classes* is based on probabilities and objective and rigorous fit indices. Unlike other data-driven approaches, such as cluster analysis, that assign an individual to one group only, LPA examines the probability of membership to each cluster or class. Thus, LPA can inform the definition of mutually exclusive taxonomies with a greater level of certainty. Another advantage of LPA is that it handles missing data, unlike other statistical approaches. In clinical studies, missing data are common and often unavoidable; however, missingness is often not reported.^28,29^ In the phenotyping literature, there are no studies to date that have reported or examined the impact of missing data on the derived phenotypes. In our own prior studies, we excluded patients with missing data, given that cluster analyses cannot handle missing data. As such, LPA offers an opportunity to (i) include patients with missing data and (ii) systematically examine the impact of missing data.

First, we test several models and use a variety of fit indices to derive the most meaningful model. Second, we test the added value of replacing missing data and examine factors that may contribute to missingness. Finally, we examine the demographic and clinical profiles of the cognitive phenotypes. Epilepsy syndromes offer an opportunity to examine methods of cognitive phenotyping as they represent a neurological condition with both focal and generalized pathology, thus providing insight into brain–behaviour relationships within phenotypes. As such, the information gained from this study can be applied to other neurological conditions that may have underlying cognitive phenotypes.

## Materials and methods

### Participants

This study was approved by the institutional review boards at UC San Diego, UC San Francisco, the University of Wisconsin-Madison, the Cleveland Clinic, Emory University, Columbia University and New York University. Informed consent was collected from patients at UC San Diego, UC San Francisco, Emory University, Columbia University and the University of Wisconsin-Madison. At the Cleveland Clinic and New York University, data were collected as part of IRB-approved data registries. Patients were included in the study if they had a diagnosis of TLE, including unilateral and bilateral TLE, by a board-certified neurologist with expertise in epileptology, in accordance with the criteria defined by the International League Against Epilepsy ,^30^ and based on video-EEG telemetry, seizure semiology and/or neuroimaging evaluation. The presence of mesial temporal sclerosis (MTS) was determined by inspection of MRI images by a board-certified neuroradiologist. Information on other types of pathologies was not systematically available across centres and therefore excluded from analyses. No patients had undergone epilepsy surgery at the time of testing. One thousand four hundred and twenty-five patients with TLE met the inclusion criteria for the study. Although LPA handles missing data, a cut-off of six out of the eight neuropsychological tests was used to minimize the number of missing data points per patient. This resulted in the inclusion of 1178 patients for the final analysis (72% = 8 tests, 24% = 7 tests, 4% = 6 tests). There were no differences in demographic or clinical variables between the included and excluded cases (all *P*-values > 0.05). The average age of the final sample was 37.76 [standard deviation (SD) = 12.14], average education was 13.94 (SD = 2.806); the sample was 57% female; self-identified race distribution was as follows: 79.6% non-Hispanic white, 9.3% non-Hispanic Black, 2.9% Asian, 0.3% Native American, 1.9% multiracial and 5.9% unknown/not reported. Approximately 2.3% of the total sample self-identified as Hispanic/Latinx.

### Neuropsychological measures

Neuropsychological testing was conducted in English without the aid of an interpreter. The following tests were common across the centres and were selected based on recommendations from the National Institute of Neurological Disorders and Stroke Epilepsy Common Data Elements(CDEs)^31^ and the International League Against Epilepsy Neuropsychology Task Force Diagnostic Methods Commission.^32^ In addition, measures of motor dexterity and processing speed were included based on previous studies demonstrating that these skills are often impaired in TLE patients with generalized impairment.^10,18^ Verbal memory was evaluated with the Wechsler Memory Scale-Third or Fourth Edition Logical Memory, immediate (LM1) and delayed recall (LM2).^33^ The CDEs recommend list learning measures to assess verbal memory; however, there was variability in the tests administered across centres and therefore, list learning was not included in this study. Language ability was evaluated with the Boston Naming Test (BNT)^34^ and letter (F–A–S) and animal fluency, measures that tap into semantic memory; mental flexibility/set-shifting was measured with the Trail Making Test B (TMT-B); processing speed was measured with the TMT-A; fine motor dexterity was measured with the Grooved Pegboard test to obtain a proxy for medication effect.^35,36^ There were limited common visuospatial tests across centres, which has been a limitation across other multicentre studies of cognitive phenotypes.^12,37^ Given that the scores for the dominant and non-dominant hands for the Grooved Pegboard test were highly correlated (*r* = 0.532, *P* < 0.001) in our sample, scores from the dominant hand (PegD) were selected to reduce collinearity. Although letter fluency (LF) has both a language and an executive function component, it showed a strong correlation with BNT (*r* = 0.395, *P* < 0.001) and animal fluency performance (*r* = 0.605, *P* < 0.001) at the TLE group level. Age-corrected scaled scores were calculated for LM1 and LM2 based on normative data provided by the test manual. Race, age, education and sex-corrected *T*-scores were calculated for the BNT, LF, animal fluency, TMT-A, TMT-B and PegD based on normative data from the expanded Halstead–Reitan Battery.^38^ All scores were converted to *T*-scores for interpretability. The distribution of missing data across tests was: animal fluency (*n* = 209), LF (*n* = 63), PegD (*n* = 39), TMT-B (*n* = 24), BNT (*n* = 13), TMT-A (*n* = 8), LM2 (*n* = 6) and LM1 (*n* = 5).

### Statistical analysis

#### Latent profile analysis

Latent profile analysis was conducted using Mplus Version 8.^39^ The following continuous variables were included in the model: LM1, LM2, BNT, animal fluency, LF, TMT-A, TMT-B and PegD. As a *post hoc* analysis, we included centre as a categorical indicator in the model to control for centre (i.e. epilepsy centre) effects on group membership. Although LPA handles missing data, the models were also evaluated with imputed data using multiple imputations in SPSS.^40,41^ Missing scores were replaced with the average score across five imputed data sets. There were 853 (73%) patients with a complete data set. There were differences in missingness across the centres [Fisher’s exact (FE) = 496.27, *P* < 0.001], with UC San Francisco, Cleveland Clinic and UC San Diego having the most missing data. There were differences in age [*t*(523.39) = 1.996, *P* = 0.023] and education [*t*(681.16) = −6.479, *P* < 0.001] between the patients with complete data and those with missing data. Patients with complete data were younger in age (mean = 37.26, SD = 12.59) and had greater years of education (mean = 14.25, SD = 2.87) relative to patients with missing data (age mean = 39.17, SD = 14.40; education mean = 13.16, SD = 2.45). However, effects sizes calculated with Cohen’s *d* were in the small range (age *d* = 0.138; education *d* = 0.394). There were no differences in the distribution of sex, age of epilepsy onset and duration of the disease (all *P*-values > 0.05).

The following model indices were evaluated to determine the optimal number of classes/profiles: Lo–Mendell–Ruben adjusted likelihood ratio test (LMRT),^42^ Bootstrapped likelihood ratio test (BLRT),^43–45^ Akaike information criteria (AIC),^46^ Bayesian information criterion (BIC),^47^ sample size-adjusted BIC^47^ and entropy.^43^ The LMRT provides an indication of statistically significant improvement by comparing the solution being evaluated with a more complex solution; a significant LMRT indicates that a more complex solution (e.g. four-class) provides a better fit relative to a less complex model (e.g. three-class). Similar to the LMRT, the BLRT statistically compares a more complex model to a less complex one by using repeated sampling methods. The AIC, BIC and size-adjusted BIC are each based on the log likelihood function for each individual model and lower values indicate better relative fit. Entropy is a measure on how well the classes/profiles can be distinguished and is calculated from the posterior probabilities. Each individual is assigned a posterior probability for each class rather than being assigned to one and only one class. Entropy is therefore the aggregate of the posterior probabilities and it ranges from zero to one, with higher values (>0.80) indicating that the classes can be highly distinguished. In addition to the indices described above, each class sample size was evaluated. The interpretability of each class was evaluated to determine if a specific class solution was consistent with previous research.

An analysis of agreement using Cohen’s Kappa statistic was performed to determine the consistency of impairment classification between missing data and imputed data. Discriminant function analyses (DFAs) were conducted to further validate the distinctiveness of the latent classes. The R3Step approach in MPlus was used to compare categorical and continuous sociodemographic and clinical variables associated with class membership.^48,49^ This approach simultaneously estimates the best-fitting solution while evaluating the associations between class membership and variables of interest, thus accounting for potential misclassification in class membership. The DCONTINUOUS command was used for continuous variables and DCATEGORICAL for categorical variables. Analyses of covariance (ANCOVAs), controlling for age, sex and education, were conducted to compare neuropsychological test performance (*T*-scores) across groups. When results from the ANCOVAs were significant, group contrasts were assessed using *post hoc* pairwise tests with the Bonferroni correction. Multiple comparisons were corrected using the Benjamini–Hochberg false discovery rate.^50^

### Data availability statement

The authors have full access to all study data and participant consent forms and take full responsibility for the data, the conduct of the research, the analyses and interpretation of the data and the right to publish all data. The data that support the findings of this study are not publicly available because of IRB-based restricted access, but further information about the data sets is available from the corresponding author on reasonable request.

## Results

### Latent profile analysis

**Figure 1:**
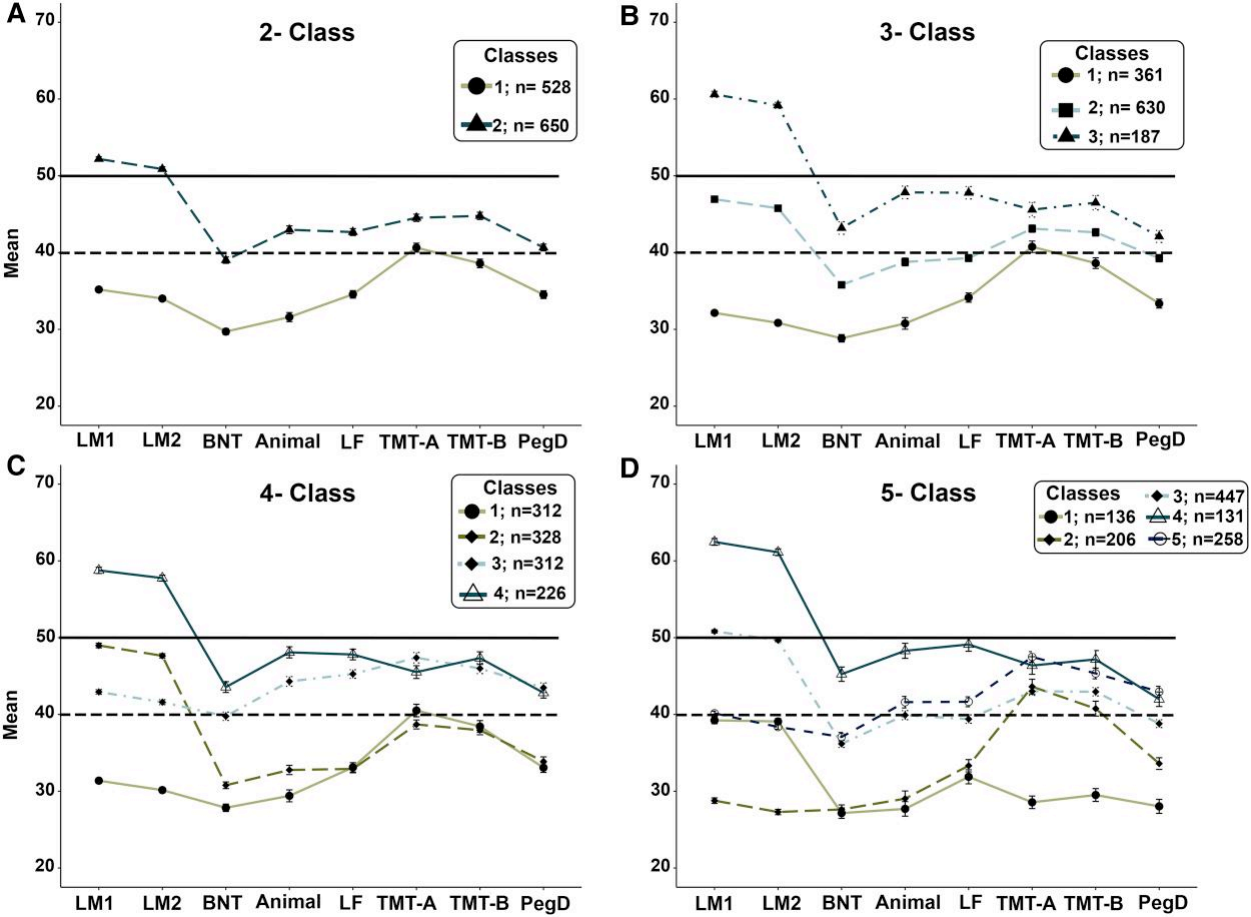
**Cognitive scores across class solutions**. Data are presented as the average *T*-scores across tests for each class. The solid line represents average normative scores, and the dashed line represents impairment at 1 SD below the mean of a healthy normative sample. (**A**) The distribution of *T*-scores for the two-class solution. A solid line with circles represents Class 1 and a dashed line with triangles represents Class 2. (**B**) The distribution of *T*-scores for the three-class solution. A solid line with circles represents Class 1, dashed line with squares represents Class 2 and dashed line with triangles represents Class 3. (**C**) The distribution of *T*-scores for the four-class solution. Solid line with circles represents Class 1, dashed line with diamonds represents Class 2, two-dashed line with diamonds represents Class 3 and solid line with triangles represents Class 4. (**D**) The distribution of *T*-scores for the five-class solution. Solid line with circles represents Class 1, dashed line with diamonds represents Class 2, two-dashed line with diamonds represents Class 3, solid line with triangles represents Class 4 and dashed line with circles represents Class 5.

Table 1 demonstrates the fit indices and sample sizes across the different class solutions for both the missing data and the imputed data. For the data set with missing data (Table 1), the best fitting and most substantively meaningful solution had three classes based on entropy, fit statistics and pattern of scores. For the three-class solution, entropy was 0.816 but dropped below 0.80 when increasing to a four-class solution; the LMRT test went from being significant with the three-class solution (*P* < 0.01) to being non-significant when moving to the four-class solution (*P* = 0.116). Figure 1 shows the pattern of impairment for each class without the imputed data. For descriptive purposes only, impairment was defined as 1 SD below the mean (*T*-score < 40). For the three-class solution, Class 1 demonstrated impairments across most tests (7/8 tests) with predominant memory and language impairments, Class 2 demonstrated predominantly impairments in language and Class 3 demonstrated no impairments at the group level with relatively high scores in memory. The models were also tested with the sample that had at least seven out of the eight tests available and the results were consistent with the above sample.

**Table 1:** Fit indices with and without missing data

	AIC	BIC	sBIC	Entropy	LMRT (*P*)	BLRT	Sample per class
Fit indices across class solutions with missing data							
2 classes	69 278.61	69 405.40	69 325.99	0.775	1269 (<0.001)	<0.001	1 = 528; 2 = 650
**3 classes**	**68 803**.**56**	**68 976**.**01**	**68 868**.**01**	**0**.**816**	**485** (**<0.001)**	**<0**.**001**	**1 = 361; 2 = 630; 3 = 187**
4 classes	68 570.14	68 788.21	68 651.63	0.737	247 (0.116)	<0.001	1 = 312; 2 = 328; 3 = 312; 4 = 226
5 classes	68 289.12	68 552.84	68 387.67	0.807	225 (0.35)	<0.001	1 = 136; 2 = 206; 3 = 447; 4 = 131; 5 = 258
Fit indices across class solutions with missing data and centre as an indicator							
2 classes	73 208.33	73 395.98	73 278.46	0.780	1321 (<0.001)	<0.001	1 = 539; 2 = 639
**3 classes**	**72 720**.**38**	**72 984**.**10**	**72 818**.**93**	**0**.**820**	**513** (**<0.001)**	**<0**.**001**	**1 = 358; 2 = 634; 3 = 186**
4 classes	72 495.02	72 834.82	72 622.00	0.799	253 (0.216)	<0.001	1 = 232; 2 = 602; 3 = 174; 4 = 170
5 classes	72 197.03	72 612.89	72 352.43	0.817	239 (0.764)	<0.001	1 = 126; 2 = 211; 3 = 269; 4 = 445; 5 = 127
Fit indices across class solutions with imputed data							
2 classes	71 859.50	71 986.29	71 906.88	0.776	1260 (<0.001)	<0.001	1 = 527; 2 = 651
3 classes	71 363.22	71 535.66	71 427.66	0.823	506 (<0.001)	<0.001	1 = 358; 2 = 638; 3 = 182
**4 classes**	**71 059**.**93**	**71 278**.**01**	**71 141**.**42**	**0**.**834**	**316** (**<0.001)**	**<0**.**001**	**1 = 217; 2 = 380; 3 = 448; 4 = 133**
5 classes	70 857.37	71 121.09	70 955.92	0.807	217 (0.23)	<0.001	1 = 138; 2 = 207; 3 = 254; 4 = 450; 5 = 129
Fit indices across class solutions with imputed data with centre as an indicator							
2 classes	75 789.27	75 976.92	75 859.39	0.780	1312 (<0.001)	<0.001	1 = 546; 2 = 632
**3 classes**	**75 274**.**58**	**75 538**.**30**	**75 373**.**13**	**0**.**825**	**540** (**<0.001)**	**<0**.**001**	**1 = 353; 2 = 643; 3 = 182**
4 classes	75 049.60	75 389.39	75 176.58	0.801	253 (0.884)	<0.001	1 = 214; 2 = 606; 3 = 188; 4 = 170
5 classes	74 759.47	75 175.34	74 914.87	0.816	233 (0.760)	<0.001	1 = 127; 2 = 214; 3 = 261; 4 = 450; 5 = 126

Bold signifies the most optimal solution.

AIC, Akaike’s information criterion; BIC, Bayesian information criterion; sBIC, size-adjusted-Bayesian information criterion; LMRT, Lo–Mendell–Ruben adjusted likelihood ratio test; BLRT, bootstrapped likelihood ratio test.

As a *post hoc* analysis, centre (i.e. epilepsy centre) was included as an indicator to control for potential effects of centre. Table 1 includes the values across the class solutions after controlling for centre for the raw data. The three-class solution continued to be the most meaningful solution. To examine if group membership changed after controlling for centre, Cohen’s Kappa statistic was examined between class solutions with and without centre as an indicator. There was almost perfect agreement for the two-class (κ = 0.961, *P* < 0.001; 98.04% concordance rate) and three-class solutions (κ = 0.963, *P* < 0.001; 97.79% concordance rate). Agreement was minimal for the four-class solution (κ = 0.368, *P* < 0.001; 53.26% concordance rate), and there was no agreement for the five-class solutions (κ = 0.112, *P* < 0.001; 28.61% concordance rate).

**Figure 2:**
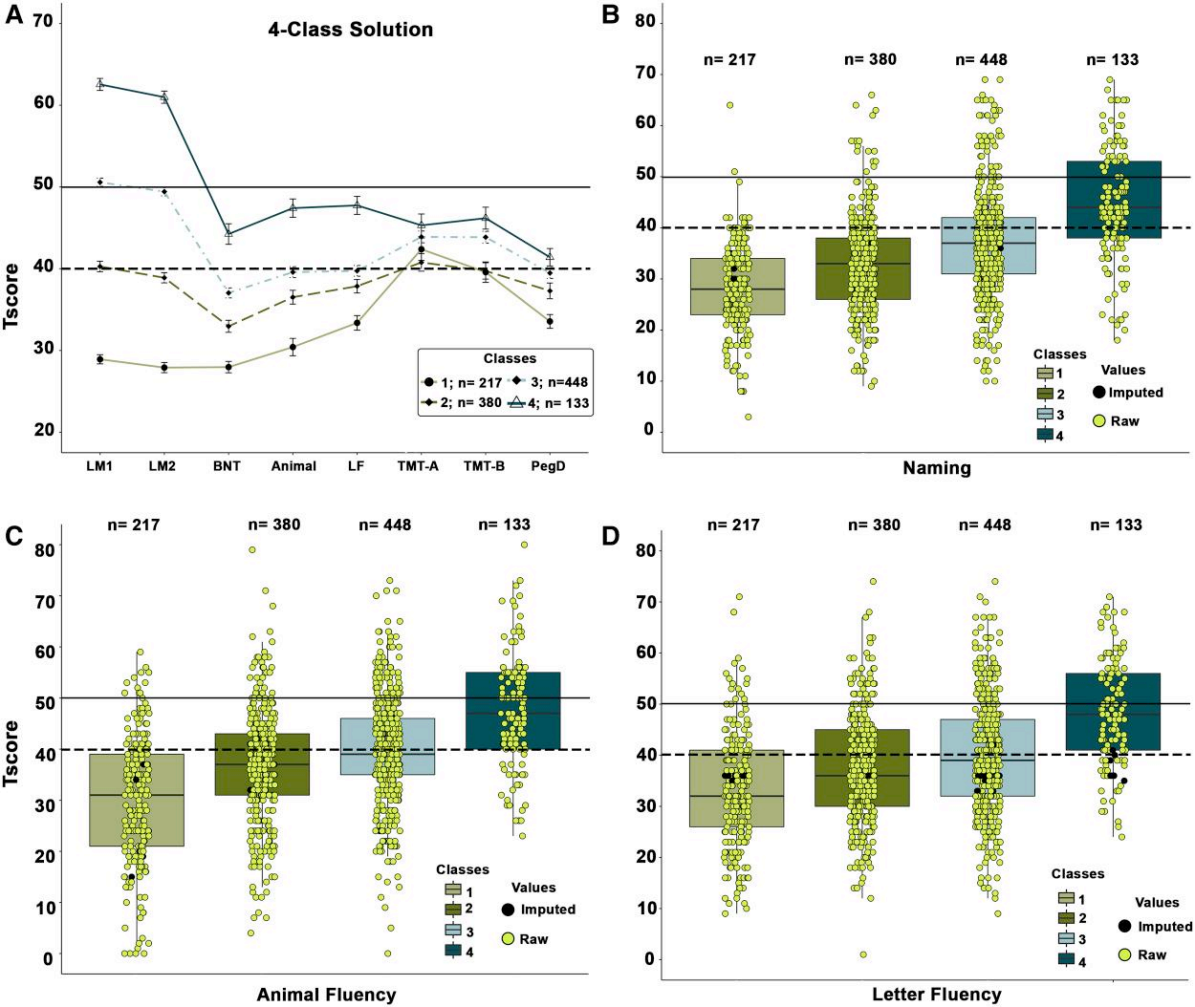
**Class 4 solution with imputed data.** (**A**) Data are average *T*-scores across tests for each class utilizing the imputed data for the four-class solution. The solid line represents average normative scores and the dashed line represents impairment at 1 SD below the mean of a healthy normative sample. (**B–D**) Distribution of *T*-scores for measures of language for each class within the 4-class solution. Data are shown for (**B**) naming (BNT), (**C**) animal fluency and (**D**) LF. The raw and imputed data points are represented as dots. The solid line represents average normative scores and the dashed line represents impairment at 1 SD below the mean of a healthy normative sample.

For the imputed data set (Table 1), the four-class solution was the best fitting given that entropy was the highest and the LMRT was significant when moving to a four-class solution from a three-class solution, but non-significant when moving to a five-class solution. Figure 2A shows the pattern of impairment for each class across the four-class solution based on the imputed data. Class 1 demonstrated impairment across most tests (7/8) with predominant deficits in memory and language; Class 2 demonstrated impairments in language and borderline impairments in delayed memory; Class 3 showed mainly impairments in naming (BNT); Class 4 had an overall intact profile. Fine motor dexterity was impaired across Classes 1–3. Given that the language measures had the most missing data, the distribution of scores was plotted for BNT (Fig. 2B), animal (Fig. 2C) and LF (Fig. 2D), with individual data points coded by whether they were raw values or imputed values.

Again, as a *post hoc* analysis, centre was included as an indicator for the imputed data set. Table 1 includes the values across the class solutions after controlling for site. The three-class solution was the most meaningful solution after controlling for centre. Cohen’s Kappa statistic revealed almost perfect agreement for the two-class solution (κ = 0.947, *P* < 0.001; 97.36% concordance rate), three-class solution (κ = 0.958, *P* < 0.001; 97.54% concordance rate) and the five-class solution (κ = 0.958, *P* < 0.001; 96.85% concordance rate). There was low agreement for the four-class solution (κ = 0.029, *P* < 0.001; 29.71% concordance rate).

### Agreement between missing data and imputed data

Cohen’s Kappa statistic revealed an almost perfect agreement between the data set with missing data and the imputed data for the two-class (κ = 0.985, *P* < 0.001; 99.23% concordance rate) and three-class (κ = 0.983 *P* < 0.001; 98.98% concordance rate) solutions. A weak agreement was found for the four-class (κ = 0.288, *P* < 0.001; 47.37% concordance rate) and the five-class (κ = 0.120, *P* < 0.001; 28.9% concordance rate) solutions. Further examination of the four-class solution demonstrated that misclassification was mostly between Class 2 (49% misclassified as Class 3 with the imputed data) and Class 3 (50% misclassified as Class 2 with the imputed data). Subsequent analyses were conducted on the data set with missing data points and without centre as indicator to demonstrate the utility of LPA with handling missing data.

### Probability of group membership

**Figure 3:**
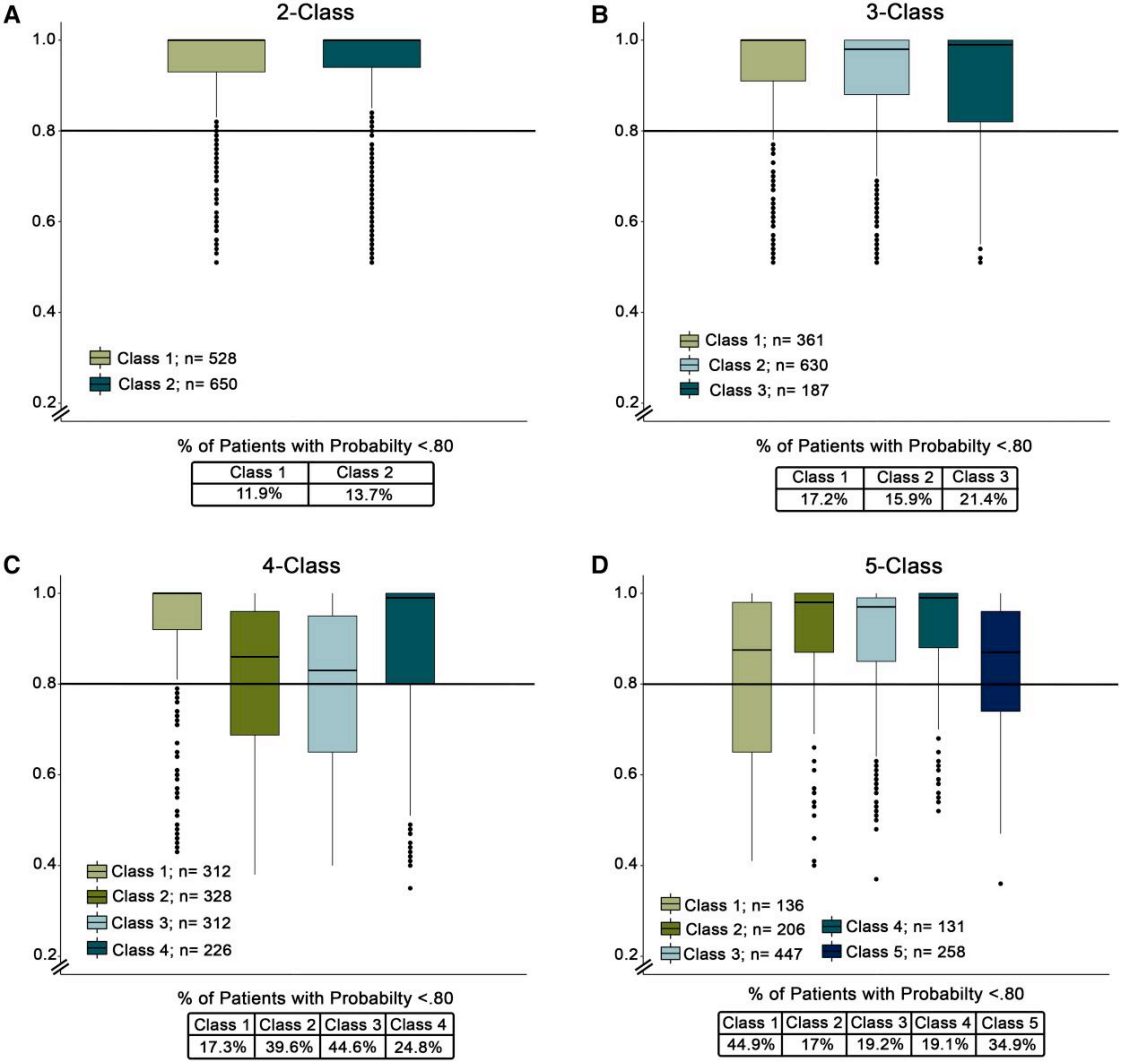
**Distribution of probability of group membership.** (**A**) Data represent the distribution of probability of group membership for the two-Class solution. The solid line represents a probability of group membership below 0.80 with 11.9% of Class 1 and 13.7% of Class 2 having a probability below 0.80. (**B**) Data represent the distribution of probability of group membership for the three-class solution. Approximately 12.2% of Class 1, 15.9% of Class 2 and 21.4% of Class 3 had a probability below 0.80. (**C**) Data represent the distribution of probability of group membership for the four-class solution. Approximately 17.3% of Class 1, 39.6% of Class 2, 44.6% of Class 3 and 24.8% of Class 4 had a probability below 0.80. (**D**) Data represent the distribution of probability of group membership for the five-class solution. Approximately 44.9% of Class 1, 17% of Class 2, 19.2% of Class 3, 19.1% of Class 4 and 43.9% of Class 5 had a probability below 0.80.

Figure 3 shows the distribution of probability of group membership for each class solution. The average probabilities were as follows: two-class (mean = 0.933, SD = 0.121; range = 0.51–1); three-class (mean = 0.914, SD = 0.129; range = 0.51–1); four-class (mean = 0.848, SD = 0.173; range = 0.35–1); five-Class (mean = 0.877, SD = 0.149; range = 0.36–1). A cut-off of above 80% was considered good probability of group membership. The percentage of patients with a probability <80% was the lowest for the two-class solution (12.8%), followed by three-class (17.1%), five-class (24.9%) and highest in the four-class solution (32.2%).

### Discriminant function analysis

**Figure 4:**
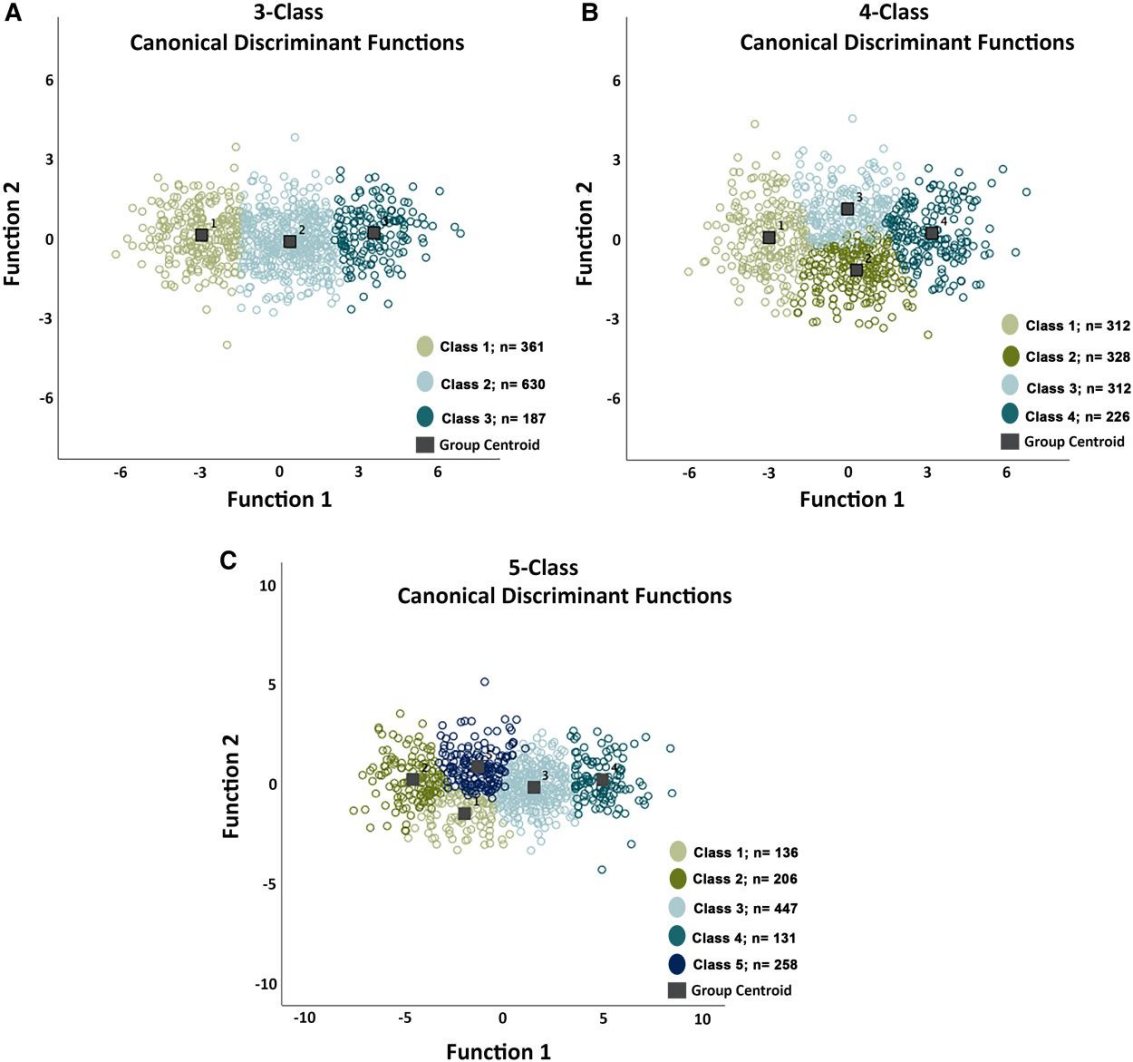
**Scatterplot of canonical DFA.** (**A**) Scatterplot of the canonical DFA for the Class 3 solution. The DFA correctly classified 96.2% of the cases. (**B**) Scatterplot of the canonical DFA for the Class 4 solution. The DFA correctly classified 95.9% of the cases. (**C**) Scatterplot of the canonical DFA for the Class 5 solution. The DFA correctly classified 95.8% of the cases.

To further validate the distinctiveness of the latent classes, DFA was performed with the cognitive scores as predictors of latent class membership. The DFA indicated that 97.9% of cases were correctly classified in the two-class solution; 96.2% in the three-class solution; 95.9% in the four-Class solution; 95.8% in the 5-Class solution. Figure 4 shows the scatter plots of individuals on the discriminant dimensions for the three-, four- and five-class solutions.

Based on the fit indices, posterior probability and distinctiveness of the latent classes, a three-class solution was selected. This was further supported by the patterns of cognitive impairment observed, which were similar to what has been reported in prior literature on cognitive phenotypes in TLE.^10–13,18,19^ As described, patients in class 1 demonstrate a profile characterized by impairments across most tests with prominent impairments in verbal learning, memory and language and are labelled the *Multidomain* Phenotype hereafter. Class 2 showed a predominantly language impaired profile and will be labelled *Language* Phenotype hereafter. Patients in Class 3 showed a profile characterized by no measurable impairments across tests and are labelled with the *No Impairment* Phenotype.

### Differences in demographics, clinical and neuropsychological variables

Table 2 shows differences in demographic and clinical variables across phenotypes for the 3-class solution and Table 3 shows the follow-up group contrasts. There were differences in age, education, age at onset of epilepsy, disease duration and number of anti-seizure medications (ASM) across phenotypes. The *Multidomain* group had a younger age, fewer years of education, a younger age of epilepsy onset and a longer disease duration relative to the *Language Impaired* and the *No Impairment* phenotypes. The *Multidomain* phenotype also had a greater number of ASMs relative to the *No Impairment* phenotype. The *Language* phenotype had a younger age, fewer years of education, younger age of epilepsy onset, a longer duration and a greater number of ASM relative to the *No Impairment* phenotype. There were differences in the presence of MTS, with the *No Impairment* group having fewer patients with MTS (26.4%) relative to the *Multidomain* (38.5%) and *Language* phenotype (35.9%). There were no other differences across phenotypes. Although there were no differences in side of seizure onset across the phenotypes, given that the overall proportion of patients with left TLE was higher across all groups, we conducted *post hoc* analyses comparing language scores across side of seizure onset within the *Language* phenotype. There were differences in BNT scores *F* (2596) = 9.12, *P* < 0.001, with bilateral TLE (*P* = 0.024; mean = 34.22, SD = 12.65) and left TLE (*P* < 0.001; mean = 34.61, SD = 9.22) having lower scores compared with right TLE (mean = 38.32, SD = 10.35). There were no differences in animal fluency: *F* (2, 464) = 0.096, *P* = 0.908 or LF *F* (2, 565) = 0.966, *P* = 0.381. Despite the differences in BNT scores, there were no differences in the probability of group membership across sides of seizure onset: *F* (2, 598) = 0.920, *P* = 0.399.

**Table 2:** Clinical and demographic characteristics across phenotypes for the 3-class solution

	Multidomain	Language	No impairment	*P*-value
*N*	361	630	187	
Per cent of sample	30	53	16	
Age	35.56 (0.66)	38.13 (0.53)	40.57 (0.96)	**<0.001**
Education	12.84 (0.13)	13.93 (0.10)	15.84 (0.23)	**<0**.**001**
Age of onset	15.14 (0.65)	20.76 (0.61)	26.63 (1.22)	**<0**.**001**
Duration (years)	20.37 (0.76)	17.44 (0.54)	13.69 (0.86)	**<0**.**001**
Current # of ASMs^a^	2.19 (0.06)	2.07 (0.05)	1.79 (0.07)	**<0**.**001**
Sex	–	–	–	0.317
Male	163 (45%)	264 (42%)	80 (43%)	
Female	198 (55%)	366 (58%)	107 (57%)	
Handedness				0.267
Left	46 (13%)	77 (12%)	26 (14%)	
Right	309 (86%)	535 (85%)	154 (82%)	
Ambidextrous	5 (1%)	16 (3%)	7 (4%)	
MTS				**0**.**040**
Yes	124 (39%)	199 (36%)	39 (26%)	
No	198 (61%)	355 (64%)	109 (74%)	
Onset side	202/107/40	365/182/54	113/46/15	0.431
Left	202 (58%)	365 (61%)	113 (65%)	
Right	107 (31%)	182 (30%)	46 (26%)	
Bilateral	40 (11%)	54 (9%)	15 (9%)	

Bold signifies the most optimal solution.

ASM, anti-seizure medications. Standard errors are presented inside the parentheses. Bold signifies statistical significance using the R3Step approach.

^a^ More than 20% of data are missing.

**Table 3:** Group contrasts for demographic and clinical variables using R3Step approach

	Multidomain versus language	Multidomain versus no impairment	Language versus no impairment
Age	0.002	<0.001	0.025
Education	<0.001	<0.001	<0.001
Onset	<0.001	<0.001	<0.001
Duration	0.002	<0.001	<0.001
Current # of ASMs	0.155	<0.001	0.002
MTS	0.655	0.015	0.025

ASM, anti-seizure medications; MTS, mesial temporal sclerosis.

There were differences across all neuropsychological measures (Table 4). Group contrasts revealed significant differences between phenotypes for all tests except for TMT-A. For TMT-A, there were no differences between the *Language* and *No Impairment* groups (*P* = 0.079). Cohen’s *d* effect sizes were calculated to determine the difference in magnitude between groups (Table 5). Effect sizes between groups ranged from small to large and the pattern of effect sizes was consistent across groups.

**Table 4:** Neuropsychological differences across phenotypes for the Class 3 solution

	Multidomain	Language	No impairment	ANCOVA	*P*-value
LM1	**32.13 (6.15)**	46.95 (5.49)	60.61 (5.48)	1445.94	<0.001
LM2	**30.85** (**6.29)**	45.76 (5.55)	59.17 (5.51)	1387.49	<0.001
BNT	**28.79** (**9.09)**	**35.79** (**9.88)**	43.20 (11.03)	135.43	<0.001
Animal fluency	**30.71** (**13.1)**	**38.78** (**11.4)**	47.84 (10.8)	108.21	<0.001
LF	**34.14** (**11.2)**	**39.28** (**10.9)**	47.79 (10.6)	69.53	<0.001
TMT-A	40.74 (14.1)	43.12 (11.9)	45.58 (13.2)	7.82	<0.001
TMT-B	**38.82** (**13.1)**	42.63 (12.1)	46.50 (12.6)	28.09	<0.001
PegD	**33.34** (**10.8)**	**39.26** (**11.7)**	42.10 (10.6)	36.75	<0.001

Estimated marginal means and standard error with covariates of education, age and sex. For descriptive purposes, bold signifies average *T*-score below the impairment cut-off (<40). Covariates: age, education, sex. Bold signifies the most optimal solution.

LM1, logical memory immediate recall; LM2, logical memory delayed recall; BNT, Boston Naming Test; LF, letter fluency; TMT-A, Trail Making Test condition A; TMT-B, Trail Making Test condition B; PegD, grooved pegboard dominant hand.

**Table 5:** Cohen’s *D* effect sizes across phenotypes

	Multidomain versus language	Multidomain versus no impairment	Language versus no impairment
LM1	2.58	4.80	2.49
LM2	2.56	4.70	2.42
BNT	0.73	1.46	0.73
Animal fluency	0.67	1.39	0.81
LF	0.47	1.25	0.78
TMT-A	0.18	0.35	0.20
TMT-B	0.32	0.61	0.48
PegD	0.52	0.82	0.41

Cohen’s *D* effect sizes: small, 0.2; medium, 0.5; large, 0.8.

LM1, logical memory immediate recall; LM2, logical memory delayed recall; BNT, Boston Naming Test; LF, letter fluency; TMT-A, Trail Making Test condition A; TMT-B, Trail Making Test condition B; PegD, grooved pegboard dominant hand.

## Discussion

This study utilized a robust and rigorous statistical method to derive cognitive phenotypes in a large, multi-site study of 1178 patients with TLE. The major goals of the study were to adjudicate among published findings that have produced variable results and to arrive at ataxonomy of neuropsychological status in this common and problematic epilepsy syndrome. First, we found that the three-phenotype model was the most meaningful based on several fit indices and patterns of impairment; it was the most robust to missing data; the demographic and clinical profiles were consistent with prior literature. Second, we demonstrated the importance of examining the factors associated with missing data and determined whether different phenotype models are robust to the missingness. Third, we provide methods for examining the stability of the phenotypes, including examining the probability of group membership provided by LPA. As the cognitive phenotyping approach continues to gain traction in the neuropsychology literature, utilizing rigorous, person-centered methods such as LPA will inform the generalizability of the phenotypes and the translation of the cognitive phenotypes into clinical diagnostic criteria.

### Determining the optimal solution

An advantage of LPA is that individuals are assigned to classes based on membership probabilities estimated directly from the model.^26^ Further, LPA provides several fit indices that can help the researcher determine the optimal solution with a greater level of certainty. In our study, we tested five solutions (two to five classes) based on prior literature with and without imputed data. Based on the fit indices described above, the three-class solution was the optimal solution with the raw data set. As a *post hoc* analysis, we also examined the impact of the centre (i.e. epilepsy centre) on group membership and found that the centre did not significantly impact group membership for the three-class solution; however, this was not the case for the four- and five-class solutions. Therefore, studies utilizing multicentre data should examine the impact of centre on group membership by adding site as an indicator when using LPA. We also examined the posterior probabilities, which provide information on the probability of an individual belonging to the group to which they were assigned. We found that for models with multiple classes (e.g. four- and five-class), the probability of group membership decreases. In fact, for the four-class solution, ∼4% of the sample had a probability of group membership below 50% and Classes 3 and 4 within this model had a large proportion of patients with a poor probability of group membership. This may suggest that with finer characterization of phenotypes (e.g. domain-specific), it is more difficult to distinguish the groups as individual patients may have overlapping features across classes. Given that we had a limited number of tests per cognitive domain, it is possible that with a more comprehensive battery (i.e. more tests per domain) or potentially more sensitive measures (i.e. list learning instead of story recall), LPA will be able to classify individuals with a greater level of certainty. We also used DFA to further examine the distinctiveness of the groups and again we found that the correct classification using the cognitive scores only was lower for the four- and five5-class models. Overall, this suggests that in order to further divide patients into finer subgroups using data-driven approaches (i.e. verbal memory only, language only), large samples with comprehensive batteries of tests may be required. Notably, using clinical criteria may allow for the characterization of finer groups such as the single-domain impaired phenotype described in harmonized, actuarial approaches, such as the International Classification of Cognitive Disorder in Epilepsy (IC-CoDE) framework.^37,51^ Importantly, the lack of consensus regarding a standard test battery, both nationally and internationally, may be contributing to the variability and lack of stability of domain-specific cognitive patterns. Given that our sample included patients with varying underlying TLE aetiologies, another possibility is that samples with more constrained aetiological groups might show more stability and uniformity in domain-specific profiles.

### The impact of missing data

Given the nature of clinical research, missing neuropsychological data are often unavoidable. However, missing data may lead to bias and loss of information when utilizing data-driven approaches and this is particularly important for the phenotyping literature as groups are derived based on the data that are available.^29,52^ Missingness can either be (i) missing completely at random (MCAR), (ii) missing at random (MAR) or (iii) missing not at random (MNAR). Importantly, MAR is a more realistic assumption than MCAR^29^ and LPA assumes that the data are MAR. Although it is difficult to determine if neuropsychological data are missing at MAR or MNAR, given that there is no statistical test to examine this, systematically examining the characteristics of the samples may provide valuable information and inform the generalizability of the findings. In our sample, there were no significant differences in demographic and clinical variables between the final sample (*N* = 1178) and the patients that were excluded due to missing a substantial amount of data (*N* = 247). When examining the final sample, the patients with incomplete data had fewer years of education and were older in age. Notably, although this was statistically significant, the magnitude of the difference was quite small. Interestingly, *post hoc* analysis revealed differences in the proportion of patients with incomplete data across the three-class solution (FE = 14.44, *P* ≤ 0.001), with the *No Impairment* phenotype having fewer patients with missing data (17%) relative to the *Multidomain* (30%) and *Language* (30%) phenotypes. Therefore, it is possible that older age and fewer years of education were contributing factors to the missing data, or that greater cognitive impairment led to incomplete testing. Although it is not possible to determine whether these factors truly explain the missing data in our sample, this suggests that the data are not MCAR and that there may be factors (e.g. patient- or study-specific) explaining the missingness. Therefore, future studies in cognitive phenotyping should examine contributing factors to missing data, given their potential impact on the generalizability of the phenotypes.

Unlike cluster analysis, which cannot handle missing data, missing data in latent class indicators are generally acceptable in LPA. To address any pitfalls in our analyses, we replaced missing values with values imputed from the data that were available and ran the models with the imputed data sets. Results from these analyses suggested that the four-class solution was the most meaningful solution. The groups in this solution were less distinct based on the clinical interpretation of their cognitive profiles. Based on prior literature,^12,18^ the pattern of impairment with four groups or more is less consistent across studies and this may be due to the number and type of tests selected, the degree of cognitive impairment across patient samples and the method used to derive the phenotypes. Further, there was perfect agreement between the missing and imputed data sets for the two- and three-class but weak agreement for the four- and five-Class solutions. Further examination of the four-class solutions demonstrated that misclassification was most common between Classes 2 and 3, which shared similar features in their cognitive profiles. Thus, the imputed data had a greater impact when deriving finer characterizations of cognitive phenotypes and thus, future studies must consider the impact of missing data and the methods for replacing the missing data when examining more than three phenotypes. Lastly, these findings suggest that the three cognitive phenotypes described across several studies are relatively stable and are more robust to missing data compared with models with four or more classes.

### Optimal solution

Similar to prior studies,^3,10,12,13,15^ the three-class model consisted of a group of patients with multidomain impairments (30%), a sizable group with focal deficits in language (53%) and a third group with a relatively intact cognitive profile (16%). The proportion of patients in the *Multidomain* and *Language* phenotypes fell within the range reported in the literature for generalized impairment (9–29%) and focal deficits (24–54%).^3^ Surprisingly, the *No Impairment* group was relatively smaller compared with other investigations reporting 27–54% of their samples with intact profiles. Most recently, the cognitive phenotyping literature has informed the development of the IC-CoDE initiative, aimed at developing a consensus-based classification system for cognitive disorders in epilepsy research.^51^ The IC-CoDE leveraged results from the cognitive phenotyping and neuropsychology literature more broadly, to develop a framework for diagnostic decisions that utilizes the *number* of impaired domains to derive cognitive phenotypes. This framework includes four cognitive phenotypes: (i) generalized impairment (i.e. three more domains impaired), (ii) bi-domain, (iii) single-domain and (iv) cognitively intact.^37^ However, given that the initial purpose of the IC-CoDE was to provide a framework for research, more rigorous methods and external validation will be needed to determine its clinical utility and LPA provides a promising methodology to achieve this goal.

A major interest in the phenotype literature is relating the derived clusters or classes to sociodemographic and clinical variables, neural correlates and treatment outcomes. We used a robust method to examine differences in demographic and clinical variables across phenotypes, which reduces bias by accounting for the uncertainty of the best fitting class solution.^48^ These analyses revealed that the *No Impairment* phenotype had more years of education, which has been shown to serve as a protective factor against epilepsy-related pathology.^53,54^ This group also demonstrated less disease burden relative to the other two groups, including less duration of disease, fewer ASMs and fewer patients with MTS. All of these factors have been associated with an increased risk of cognitive impairment.^3,4,55^ Thus, this smaller subgroup of patients in our sample may represent a group with a combination of protective factors and less disease burden. Notably, our sample consisted of mostly drug-resistant TLE, which based on the epilepsy literature is associated with poorer cognitive profiles than those who are drug-responsive.^3,4^ However, given that most neuropsychological studies in epilepsy aggregate all patients into one group, patients with drug-resistant epilepsy, but with intact cognitive profiles have not been well characterized until recently.

Further, the *Multidomain* group is another unexpected phenotype based on the lesion model that has been hypothesized to represent a group of patients with potential co-morbid non-epilepsy pathology, elevated health-related risk factors, greater generalized tonic-clonic seizures or lower brain reserve.^3^ In our study, this group had fewer years of education, younger onset of epilepsy, a longer duration of disease, was taking more ASMs and had a greater proportion of patients with MTS. Other studies have also found that phenotypes with generalized impairment have fewer years of education,^15,18^ younger age of onset,^12,15,18^ longer disease duration,^10^ were taking more ASMs,^10^ and had greater portion of patients with MTS.^15^ In more benign forms of TLE,^13^ patients with multidomain impairments had fewer years of education as did their parents, which has been suggested to be a potential socioeconomic indicator. Finally, the *Language* phenotype also demonstrated greater disease burden relative to the *No Impairment* phenotype. It is noteworthy to mention that there were no differences in the side of seizure onset across the phenotypes, which has been a consistent finding across studies.^10–12,15^ Although this may at first appear surprising, it complements a growing literature that demonstrates a pattern of bilateral and often widespread brain abnormalities in patients with drug-resistant TLE, likely leading to a ‘non-lateralized’ pattern of impairment even in patients with a unilateral seizure onset. This again highlights how a simple lesion model fails to capture the complexity of cognitive impairments experienced by patients with TLE and lends support for a network-based approach. However, we did not have information on hemispheric language dominance and therefore could not determine if patients had epilepsy in the dominant hemisphere, which warrants further investigation. We also did not include non-verbal tests (i.e. visuospatial and visual memory), which could have contributed to the lack of laterality findings. Lastly, it is possible that our tests of language lack the sensitivity to capture subtle lateralizing deficits in language (i.e. those that would reveal greater deficits in patients with left or language-dominant TLE) or that there are other factors (e.g. number and type of ASM, bilingualism) explaining the language deficits in patients with non-dominant hemisphere epilepsy.

When examining the extent of the cognitive impairments, differences among the three groups were greater in the areas of memory and language, regardless of group membership. In fact, patients in the *No Impairment* group had scores in immediate and delayed memory that were ∼1 SD above the mean of a healthy normative sample. Although this group had the least number of patients with MTS, we tested memory with prose recall, which has been shown to be less sensitive to memory impairments relative to list learning.^37^ In the IC-CoDE application study, the base rates of impairment ranged from 22 to 24% for prose recall (i.e. LM1 and LM2) but were higher for list learning and memory (27–43%) depending on the test and impairment threshold used. Thus, it is possible that the high scores in the *No Impairment* group reflect the lower sensitivity of prose recall in detecting memory impairments in TLE. Furthermore, it is possible that finer phenotypes could emerge with the use of more sensitive tests, by considering specific test indices (e.g. recognition scores for memory, reaction times for naming), or by further deconstructing test impairment patterns (e.g. impact of ASMs).

The *Multidomain* phenotype had impaired scores in language tests that were lower than the *Language* phenotype, suggesting that this group represents patients with more pervasive impairment that may be explained by factors beyond epilepsy-related pathology. Although the *Multidomain* group had fewer years of education, a younger age at onset and a longer disease duration, which can, in part, explain the differences in cognitive scores, these differences were also found between the *Language* and the *No Impairment* phenotypes. These findings highlight the need to also explore other non-epilepsy factors that may contribute to different cognitive profiles, including both risk and protective factors. The pattern of impairment for the *Language* phenotype was surprising, given that focal or domain-specific phenotypes have been described to have impairments in both memory and language.^3,12^ Although we did not have comprehensive EEG data, information on other types of pathology or detailed ASM information available, this group of patients may represent a group with greater pathology in the lateral temporal lobe or those taking ASMs known to affect language function such as topiramate or zonisamide.^56^ Furthermore, it is possible that due to the lower sensitivity of prose recall (Logical Memory), we did not capture many patients with both language and more subtle memory impairments within this focal group. Importantly, although there were no differences in side of seizure onset across the phenotypes, patients with left TLE and bilateral TLE had lower BNT scores within the *Language* phenotype compared with right TLE. Despite these differences, patients with the right TLE within the *Language* phenotype did not have a lower probability of group membership compared with the other two groups.

Interestingly, naming had the lowest scores across all three groups regardless of the level of impairment, which is consistent with findings in the IC-CoDE validation study, which included a subset of the patients from this study.^37^ In the IC-CoDE study, deficits in BNT were the most commonly observed, with 53–67% of the patients demonstrating impairments depending on the impairment cut-off applied. Lastly, the pattern of scores across tests of processing speed, mental flexibility/set-shifting and fine motor dexterity was similar across groups, contributing less to the distinctiveness of the phenotypes.

### Strengths and limitations

This study represents the first and largest investigation of cognitive phenotypes in TLE utilizing LPA. We provide a detailed description of LPA and apply additional statistical tests that investigators in this area can use to validate the stability of cognitive phenotypes in other neurological disorders. We also examined the utility of different metrics provided by LPA, which can inform future studies in cognitive phenotyping across the neuropsychology literature. Lastly, we explore the missing data in our sample, as this could have an impact on the development and applicability of cognitive phenotypes.

Nonetheless, there are several limitations to our study that should be addressed in future investigations. First, given the multicentre aspect of our study, we had a limited number of tests per domain and did not include tests in the visual memory and visuospatial domains. The lack of visual memory and visuospatial tests has been a limitation across many studies in epilepsy phenotyping given (i) variability across tests given within these domains, (ii) poor sensitivity of these tests in detecting right hemisphere and right medial temporal lobe dysfunction^57–59^ and (iii) base rates of impairment across these domains that are lower relative to other domains. The application of the IC-CoDE, which includes a subset of the patients from this study, included a visuospatial domain and demonstrated that this domain was less commonly available across six major epilepsy centres in the USA, with many cases missing visuospatial data. Furthermore, the visuospatial domain was the least impaired across a sample of 2485 patients with drug-resistant TLE and only 1–2% of the single-domain phenotype had an isolated visuospatial impairment. Regarding visual memory, studies that have included visual memory tests^10,18,37,60^ have combined verbal and visual memory tests into one domain and it remains to be determined if an isolated visual memory phenotype exists. A recent meta-analysis explored the lateralizing capabilities of non-verbal memory tests in patients with unilateral TLE and noted that the stimulus type (e.g. designs, faces, objects), learning format (i.e. single, repeated), test delay (i.e. learning delayed) and test format (i.e. recall, recognition) have varying degrees of sensitivity in detecting lateralizing effects.^61^ Thus, we first need consensus as a field on the most sensitive visual memory tests to use, as well as a deeper understanding on the psychometric properties of these tests^62^ and how they will impact the nature of the phenotypes. Nonetheless, it is possible that the *Multidomain* phenotype in our study had intact or minimally impaired visuospatial abilities, representing a phenotype with primarily verbal-based impairments.

Second, we did not include measures of list learning, which have been shown to be sensitive to medial temporal dysfunction. In the IC-CoDE study, there were differences across sites on the type of measure given for list learning, with some sites utilizing the California Verbal Memory Test (CVLT) and other sites the Rey Auditory Verbal Learning Test (RAVLT). It has been shown that standard scores for the CVLT are significantly lower relative to the RAVLT^63^ and therefore, harmonizing methods between these two tests are needed to reduce the missingness in future studies. Importantly, including a list learning test may provide greater sensitivity at detecting differences in clinical characteristics such as side of seizure onset and underlying aetiologies (e.g. MTS versus lateral temporal).

Third, a subset of the patients included in this study (34.5%; *n* = 407) were also included in our previous study, where we compared an actuarial criteria to cluster analyses.^12^ In the present study, we added three new centres (Columbia, NYU, Emory), which comprised ∼56% of the sample. Amassing large samples of well-characterized patients with epilepsy presents a challenging task in epilepsy research and one that requires multicentre collaborations and therefore, studies may have overlapping samples. Although this is not uncommon across other literatures that frequently use shared data sets, it nonetheless represents a limitation. Fourth, we excluded 247 patients due to having a significant amount of missing data. Our study demonstrated that there were some differences in demographic and clinical characteristics between patients with complete data and those missing tests. This suggests that there is a subset of patients that is not being captured in the cognitive phenotyping literature due to missing data and therefore, findings from these studies may not be applicable to this subset of patients. Fifth, the systematic exclusion of patients with intellectual disability (ID) in our study was complicated by the use of different measures for estimating intelligence quotient across centres which are not fully comparable. However, given that patients with ID are often excluded from neuropsychological studies, this could be beneficial to consider for future research.

Sixth, we did not have comprehensive information on other non-epilepsy comorbidities or language status (i.e. bilingualism) that may further explain the heterogeneity observed as proposed by other studies. Determining the impact of bilingualism on these phenotypes will be important given the heavy verbal demands of the tests used to determine the phenotypes. Finally, although our sample was somewhat diverse in terms of race/ethnicity, we did not have the power to examine the phenotypes within each group separately to determine if there are unique demographic and clinical characteristics that may explain the extent of cognitive impairment for each population. Future work in this area should validate the cognitive phenotypes in large, more racially/ethnically and linguistically diverse samples to improve the generalizability of the findings. Furthermore, the cognitive phenotypes should be examined utilizing different neuropsychological measures that test similar constructs. This would help to determine the generalizability of our findings and their international applicability. Although this study represents the first attempt at deriving a taxonomy, the limitations of our study highlight the need for studies with large, demographically diverse samples with a more comprehensive battery of neuropsychological tests. The IC-CoDE initiative will provide the ideal infrastructure to build national and international collaborations to develop and validate this taxonomy.

## Conclusion

The process of cognitive phenotyping based on heterogeneous tests is not intended to replace single or multi-cohort studies that are designed to dissect the neuroanatomy of TLE. Rather, cognitive phenotyping leads to an improved understanding of the presence and frequency of *combinations* of impairments that characterize TLE and the opportunity to determine the underlying factors that drive phenotypic membership. The cognitive phenotype approach can also help to provide a framework for large-scale collaborative efforts that will have to rely on different tests and languages and address cross-culture issues in the neuropsychology of epilepsy.

### Future directions

Cognitive phenotyping offers researchers a novel approach to uniformity, harmonization and communication regarding cognitive profiles in research. We do not propose that clinical practice or judgement be replaced with this approach. The path from research aimed at identifying reliable cognitive phenotypes to their application in clinical practice is a process that will take time. Importantly, clarification of several issues, including the reliability of identified clusters, aetiological issues, linked biomarkers (e.g. imaging, genetics), the clinical course and other characteristics are important issues to examine before these methods are implemented clinically. Furthermore, the international applicability, particularly in linguistically and ethnoracially diverse samples, is warranted before clinical translation can be accomplished. Nonetheless, initiatives such as the IC-CoDE will provide the infrastructure to move the path from research to clinical practice forward.

## Abbreviations

AIC =: Akaike information criteria
ANCOVA =: analysis of covariance
ASM =: anti-seizure medications
BIC =: bayesian information criterion
BLRT =: bootstrapped likelihood ratio test
BNT =: Boston Naming Test
CVLT =: California Verbal Memory Test
CDEs =: common data elements
DFAs =: discriminant function analyses
ID =: intellectual disability
IC-CoDE =: international classification of cognitive disorder in epilepsy
LF= =: letter fluency
LPA =: latent profile analysis
LMRT =: Lo–Mendell–Ruben adjusted likelihood ratio test
MTS =: mesial temporal sclerosis
MAR =: missing at random
MCAR =: missing completely at random
MNAR =: missing not at random
PegD =: grooved pegbord test
RAVLT =: Rey Auditory Verbal Learning Test
TLE =: temporal lobe epilepsy
TMT =: Trail Making Test
